# Aspirations to study medicine, perceptions of a good doctor, and their influence on specialty choice among medical students

**DOI:** 10.1371/journal.pone.0326266

**Published:** 2025-06-17

**Authors:** Quang Thanh Nguyen, Thuong Thi Ha Luong, Hoang Viet Nguyen, Minh Quang Ngo, Son Hoang Nguyen, Ngoc Luong Khanh Nguyen

**Affiliations:** 1 College of Health Sciences, VinUniversity, Hanoi, Vietnam; 2 Department of Pediatric Surgery, Vietnam National Hospital of Pediatrics, Hanoi, Vietnam; Kandahar University, Faculty of Medicine, AFGHANISTAN

## Abstract

**Background:**

Specialty decision-making among medical students reflects a complex interplay of motivations, evolving perceptions, and experiences during medical training. This study explores how students’ initial motivations, views on what makes a good doctor, and academic progression influence their specialty preferences.

**Methods:**

A mixed-methods study was conducted at VinUniversity, a not-for-profit medical school in Vietnam. Quantitative surveys were completed by 155 students across Years 1–4 (79.5% response rate), and 27 students participated in in-depth interviews. Quantitative data were analyzed using Wilcoxon rank-sum and Kruskal-Wallis tests. Qualitative responses underwent thematic analysis to explore identity formation and specialty choice.

**Results:**

Altruism was the most cited factor influencing specialty choice, with “desire to help people” rated highest (4.08 ± 1.14). Students’ perceptions of essential doctor qualities evolved over time. Younger students valued creativity (p = 0.035) and innovation (p = 0.022) more than seniors, reflecting a shift toward practicality in later years. Specialty preferences aligned with professional values: students favoring surgery rated work ethic (p = 0.029), collaboration (p = 0.006), and adaptability (p = 0.044) higher than those preferring internal medicine. Students inclined toward pediatrics or psychiatry prioritized empathy and communication, while those choosing surgery emphasized technical skill and decisiveness. Female students rated prestige as more influential than male students (p = 0.049). Qualitative data revealed that clinical exposure and self-reflection helped students refine their specialty choices by aligning personal traits with perceived specialty demands.

**Conclusion:**

Medical students’ specialty preferences are shaped by their initial motivations, developing perceptions of good doctor qualities, and experiences throughout training. These findings underscore the need for medical curricula and mentorship to address the evolving identity and values of students, supporting more informed and aligned specialty choices.

## Background

The career aspirations and specialty choices of medical students are influenced by a complex interplay of intrinsic motivations, external factors, and evolving perceptions of what it means to be a competent physician [1,2]. Key factors that shape career trajectories include competitiveness, work-life balance, the influence of role models, the nature of patient interactions, and the specifics of the specialty itself [1]. Research indicates that while students may enter medical school with certain career expectations, these often change as they gain clinical experience and interact with mentors and peers [1,3]. As students progress in their education, this uncertainty tends to decrease, with final-year students displaying more defined career plans, highlighting the critical role of clerkships in determining long-term career decisions [2–4]. Additionally, there are notable gender disparities, with female students frequently reporting lower career ambitions compared to their male counterparts, and psychological distress and ineffective coping strategies significantly contributing to career indecision [4,5]. A study from Germany also notes that students experiencing increased study-related stress are less likely to have high career ambitions [5].

Understanding the dynamic evolution of medical students’ career aspirations is crucial for educators and policymakers who aim to support career development and strategically distribute the medical workforce. The motivation to pursue a medical career often arises from a mix of personal passion, societal expectations, and practical factors such as job security and financial incentives. Moreover, students’ perceptions of the ideal physician significantly influence their specialty choices. While some students may value technical skills and medical knowledge highly, others might prioritize empathy, communication skills, and a patient-centered approach. These diverse values can substantially influence students’ specialty choices, steering them toward fields that align with their personal strengths and professional ideals.

Moreover, research shows that effective role models can help students affirm their values and develop essential professional skills, qualities, and behaviors—fundamental components for professionalism and sound decision-making [3,6–8]. Studies also emphasize the considerable amount of time students spend with clinical instructors in the later stages of their medical education and how they frequently revise their career plans during their clerkship rotations [1,3,8]. It is vital for educators to recognize their substantial impact on students’ career choices [8]. Both positive and negative role modeling can have profound effects, underscoring the need for medical schools to implement robust faculty development programs that promote exceptional doctor role modeling [7,8].

In Vietnam and other lower-middle-income countries, medical education is experiencing profound transformations through the implementation of innovative educational frameworks designed to cultivate well-rounded, future-ready physicians [9]. Specifically, at VinUniversity, this transformation is uniquely tailored to the local context, integrating international educational standards with local cultural and societal needs to effectively address Vietnam’s specific healthcare challenges [9]. Despite extensive research on medical students’ career choices, there remains a significant gap in understanding how the interplay of early aspirations, role model influences, and educational experiences shapes these decisions within the rapidly evolving medical education systems like Vietnam’s. This gap highlights the need for context-specific studies that provide insights into how localized adaptations of medical education frameworks influence career trajectories and specialty choices. Addressing this gap is particularly crucial for institutions like VinUniversity, where innovative educational practices are likely to have unique impacts on the professional development of future physicians.

The objectives of this study are:

1. To examine the relationship between medical students’ initial aspirations to become doctors and their ultimate specialty choices, thereby understanding how early career motivations influence long-term career trajectories.
2. To identify how medical students’ perceptions of essential doctor characteristics influence their specialty selection, providing insights into the professional qualities most valued across different specialties.
3. To analyze the evolution of medical students’ aspirations through qualitative interviews, aiming to capture how their career goals are shaped by their educational experiences and clinical exposures.

## Method

### Ethics approval and consent to participate

This study was approved ethically by VinUniversity and the Ethical Committee of the Vinmec Healthcare System under registration number 55/2024/QD-VMEC. A cover letter outlining the study’s goals, highlighting the voluntary nature of participation, and explaining the potential risks and benefits, accompanied the questionnaires (S1 File). It also included contact information for the authors. Participants were encouraged to reach out to the researchers with any inquiries about the survey or if they chose to withdraw from the study. Written informed consent was secured from all participants prior to their involvement. For confidentiality and anonymity, participants’ names and email addresses were removed after form submission, and a unique ID was assigned to each participant.

### Research design

This study adopted a mixed-methods design, incorporating both quantitative surveys and qualitative interviews to comprehensively explore the interplay between medical students’ career aspirations, their perceptions of what constitutes an exemplary doctor, and their ultimate specialty choices.

### Quantitative component

The study began with a comprehensive survey targeting all enrolled medical students across the first four academic years at VinUniversity. Using a total population sampling method, all 195 medical students from Year 1 to Year 4 were invited to participate. The survey achieved a response rate of 79.5%.

### Qualitative component

Parallel to the survey, qualitative data were gathered through semi-structured interviews with a strategically selected group of students. Purposeful sampling was used to ensure balanced representation across academic years, gender and hometown. A total of 27 students were interviewed, evenly distributed across the cohorts: six from Year 1, seven from Year 2, seven from Year 3, and seven from Year 4.

### Data collection methods

Data collection was conducted from September to November 2024. The survey was administered online via Google Forms and disseminated through institutional emails and student communication channels to ensure widespread participation. Anonymity was strictly enforced to minimize bias and maximize the honesty of the responses. Additionally, to encourage participation, weekly reminders were sent throughout the survey period.

For the qualitative interviews, participants shared their detailed experiences and perspectives. Interviews were either in-person or via online platforms, depending on availability and preference, and each lasted between 30–45 minutes. They were structured around a series of open-ended questions designed to delve into the students’ evolving aspirations and influencing factors regarding their specialty choices.

With consent, all interviews were audio-recorded for accuracy and transcribed verbatim. The transcriptions were meticulously checked for accuracy against the recordings and anonymized to maintain confidentiality. The qualitative data were then analyzed using NVivo software to conduct systematic thematic analysis.

### Survey instrument and validation

The survey instrument was carefully crafted, drawing on validated tools from prior research on medical career aspirations and specialty choices [10–16]. All items were rated on a 5-point Likert scale, where 1 represented ‘strongly disagree’ and 5 represented ‘strongly agree.’ This scoring method enabled the analysis of trends and differences in mean values across various student cohorts. To ensure the survey’s relevance and clarity, it underwent a pilot test with 10 randomly selected students and was subsequently refined based on their feedback.

The survey for this study collected comprehensive demographic data and explored the motivational factors influencing medical students’ career choices. It assessed intrinsic motivations such as personal aspirations, interest in research and teaching, personal or family health experiences, and academic success. Extrinsic motivations were related to career stability and benefits, including views of medicine as a prestigious field, job security, financial incentives, and diverse professional opportunities. Social and environmental factors included familial influences in medicine, career guidance from parents or mentors, and the impact of media. Additionally, students indicated their specialty preferences, which helped analyze the link between their career goals, perceptions of medical professionalism, and chosen specialties. The survey also categorized key characteristics of a good doctor into four domains as per established medical professionalism frameworks like those developed by the Royal College of Physicians and Surgeons of Canada (CanMEDS) or the American Board of Internal Medicine’s professionalism framework (ABIM) (Table 1) [17].

**Table 1:** Key Domains and Essential Characteristics of a Good Doctor.

Domain	Characteristics
**1. Personal and Ethical Attributes** *(Professionalism & Integrity)*	- **Accountability**: Taking responsibility for actions and decisions. - **Integrity**: Maintaining ethical standards and honesty. - **Humility**: Recognizing one’s limitations and being open to feedback. - **Self-awareness**: Understanding one’s own biases, emotions, and strengths. - **Work Ethic**: Commitment to professionalism, responsibility, and dedication to patient care.
**2. Interpersonal and Communication Skills** *(Patient-Centered Care & Collaboration)*	- **Communication Skills**: The ability to effectively interact with patients, families, and colleagues. - **Collaboration**: Working efficiently within healthcare teams. - **Empathy**: Understanding and sharing the feelings of others. - **Compassion**: Providing care with kindness and sensitivity to patient suffering.
**3. Cognitive and Problem-Solving Skills** *(Clinical Reasoning & Critical Thinking)*	- **Problem-Solving Abilities**: Diagnosing and addressing medical issues effectively. - **Adaptability**: Adjusting to new challenges, patient conditions, or healthcare advancements. - **Creativity & Innovation**: Thinking outside the box to improve patient care and medical solutions.
**4. Leadership and Resilience** *(Capacity to Lead and Sustain a Medical Career)*	- **Leadership**: Guiding healthcare teams and making crucial decisions. - **Resilience**: Coping with stress and setbacks in a high-pressure environment.

### Statistical analysis

Descriptive statistics were used to summarize demographic variables and motivational factors. Continuous variables were reported as means ± standard deviations, while categorical variables were presented as frequencies and percentages. Group comparisons were conducted using Wilcoxon rank sum tests for two-group comparisons and Kruskal-Wallis tests for multiple groups. Chi-square tests assessed associations between categorical variables. p-values were interpreted with caution due to multiple comparisons and that no formal correction (e.g., Bonferroni) was applied, as this was an exploratory study.

Ordinal logistic regression models were applied to examine factors influencing specialty choice, adjusting for demographic covariates. Multiple linear regression analyzed the impact of demographic variables on the perceived importance of professional attributes. Statistical significance was set at p < 0.05. All analyses were performed using R.

The qualitative interview data were transcribed and analyzed using NVivo qualitative analysis software, following a thematic analysis approach to explore how students’ aspirations evolved and the factors influencing their career choices throughout medical school.

## Result

### I. Quantitative analysis

#### 1. Participant characteristics.

Table 2 presents demographic and academic data for 155 medical students with the mean age of 21.29 (± 2.12) years old. The majority of students (84%) are from urban areas. The most popular specialty choice is Surgery at 39%, followed by Internal Medicine at 12%, Obstetrics and Gynecology at 8.4%, and Pediatrics at 6.5%. The remaining 35% of students are distributed across 15 other minor specialties.

**Table 2:** Demographic Data of Participants.

Variable	N = 155^*1*^
**Year Level**	
Year 4	43 (28%)
Year 3	40 (26%)
Year 2	48 (31%)
Year 1	24 (15%)
**Gender**	
Female	75 (48%)
Male	80 (52%)
**Hometown**	
Other	25 (16%)
Urban	130 (84%)
**Total Monthly Income**	
< 400 USD	6 (3.9%)
400–800 USD	14 (9%)
801–1200 USD	26 (16.8%)
1.201–2000 USD	27 (17.4%)
> 2000 USD	61 (39.4%)
Undisclosed	21 (13.5%)
**Choice of Specialty**	
Internal Medicine	18 (11.6%)
Obstetrics and Gynecology	13 (8.3%)
Pediatrics	10 (6.5%)
Surgery	60 (38.7%)
Others	54 (34.8%)

1 *n (%)*

#### 2. Factors influencing medical students’ decision to pursue medicine.

Table 3 displays the medical students’ ratings for various motivations and influences on their decision to pursue medicine. The highest average rating is for the desire to help people (4.08 ± 1.14), suggesting it’s the most significant motivation across all students. The lowest rating is for the influence of social media and movies (2.14 ± 1.21). The only motivation showing a potential statistical significance is the desire to help people with a p-value of 0.058, hinting at a potential difference in this motivation between earlier and later years.

**Table 3:** Factors Influencing Medical Students’ Decision to Pursue Medicine Across Cohorts.

Characteristic	Year 3 & 4^1^	Year 1 & 2^1^	Overall	p-value^2^
Desire to Help People	3.89 (± 1.25)	4.29 (± 0.97)	4.08 (± 1.14)	0.058
Stable Job	3.61 (± 1.31)	3.64 (± 1.28)	3.63 (± 1.29)	>0.9
Wide Range of Professional Opportunities	3.46 (± 1.23)	3.54 (± 1.19)	3.50 (± 1.21)	0.7
Prestigious Profession	3.30 (± 1.29)	3.57 (± 1.29)	3.43 (± 1.29)	0.2
Illness of Yourself or Close Family/Friend	3.46 (± 1.39)	3.38 (± 1.39)	3.42 (± 1.39)	0.7
Interest in Research and Teaching	2.76 (± 1.38)	2.97 (± 1.29)	2.86 (± 1.34)	0.3
Well-paid Job	2.87 (± 1.29)	2.86 (± 1.32)	2.86 (± 1.30)	>0.9
Academic Excellence in High School	2.80 (± 1.39)	2.89 (± 1.35)	2.84 (± 1.37)	0.7
Career Guidance	2.66 (± 1.50)	2.67 (± 1.48)	2.66 (± 1.49)	>0.9
Family Expectations	2.31 (± 1.26)	2.32 (± 1.30)	2.32 (± 1.27)	>0.9
Doctor Family Background	2.14 (± 1.44)	2.38 (± 1.66)	2.25 (± 1.54)	0.5
Social media and Movies	2.18 (± 1.23)	2.08 (± 1.20)	2.14 (± 1.21)	0.6

1 *Values represent the mean and standard deviation of responses rated on a 5-point Likert scale, where 1 = strongly disagree and 5 = strongly agree.*

2 *Wilcoxon rank sum test*

#### 3. Perceived characteristics of a good doctor.

Table 4 compares the perceptions of essential characteristics of a good doctor across cohorts. The findings demonstrate consistently high ratings across most attributes such as work ethics, problem-solving abilities, adaptability, communication skills, resilience, and integrity, with no significant differences in perception between the younger and older cohorts. Creativity and innovation are the exceptions where younger students (Years 1 & 2) rate these traits significantly higher than their senior counterparts (p-values of 0.035 for creativity and 0.022 for innovation), suggesting a shift in focus towards more structured approaches as students progress in their medical education. Attributes like collaboration, empathy, and compassion also scored highly, reflecting the emphasis on patient-centered skills in medical training.

**Table 4:** Comparison of Perceived Essential Characteristics of a Good Doctor Across Medical Student Cohorts.

Characteristic	Year 3 & 4	Year 1 & 2	Overall	p-value^1^
Work Ethic	4.64 (± 0.71)	4.67 (± 0.79)	4.65 (± 0.74)	0.5
Problem-solving Abilities	4.58 (± 0.63)	4.69 (± 0.55)	4.63 (± 0.59)	0.2
Adaptability	4.43 (± 0.80)	4.60 (± 0.64)	4.51 (± 0.73)	0.2
Communication Skills	4.47 (± 0.83)	4.56 (± 0.65)	4.51 (± 0.75)	0.8
Resilience	4.39 (± 0.92)	4.58 (± 0.69)	4.48 (± 0.82)	0.2
Integrity	4.46 (± 0.79)	4.44 (± 0.69)	4.45 (± 0.74)	0.6
Self-awareness	4.45 (± 0.84)	4.43 (± 0.69)	4.44 (± 0.77)	0.5
Empathy	4.33 (± 0.94)	4.54 (± 0.80)	4.43 (± 0.88)	0.2
Collaboration	4.39 (± 0.84)	4.47 (± 0.67)	4.43 (± 0.76)	0.8
Accountability	4.35 (± 0.79)	4.44 (± 0.79)	4.39 (± 0.79)	0.4
Compassion	4.27 (± 0.95)	4.44 (± 0.73)	4.35 (± 0.86)	0.5
Humility	4.04 (± 1.06)	4.07 (± 1.05)	4.05 (± 1.06)	0.8
Leadership	3.73 (± 1.05)	3.69 (± 1.02)	3.72 (± 1.03)	0.8
Creativity	3.41 (± 1.15)	3.81 (± 1.06)	3.59 (± 1.12)	**0.035**
Innovation	3.39 (± 1.16)	3.81 (± 1.03)	3.58 (± 1.12)	**0.022**

1 *Wilcoxon rank sum test.*

### II. Thematic analysis: Evolution of medical aspirations and its impact on career decisions

This section delves into the progression of medical students’ career aspirations during their academic journey and the resultant impact on their professional decisions. The findings can be divided into two main themes: the first theme addresses the initial motivations and aspirations that led students to pursue medicine, while the second theme examines how these aspirations evolved over time, ultimately shaping their perceptions of the medical profession and influencing their future career choices.

#### Theme 1: Initial aspirations and motivations for pursuing medicine.

Students’ motivations for choosing medicine varied widely but can be categorized into four major themes: social influence, personal motivation and interest, family background, and professional/financial considerations. These motivations shaped students’ perceptions of the medical profession before they entered medical school.

**Subtheme 1.1: Social influence: The power of peers and community:** Many students were influenced by their social environment, particularly peers and community members. Peer motivation played a crucial role in shaping aspirations, particularly for students who were surrounded by high-achieving individuals:

*I met a lot of friends, and they all had different reasons for becoming a doctor. Their goals were exciting, and it made me feel like I was on the right path.”* (Student 05, Female, Year 3) This quote highlights the impact of peer reinforcement, where exposure to ambitious and motivated peers solidified students’ initial aspirations. The perception of medicine as a noble, challenging, and intellectually stimulating career was reinforced by discussions with peers who shared similar goals. Beyond peers, students also felt a responsibility toward their community, particularly those from underprivileged backgrounds. The desire to care for others, particularly family members and vulnerable populations, was a strong motivation:

*“I chose medicine because I want to help my family and others in difficult situations.”* (Student 02, Male, Year 1).

*“Since childhood, I saw my father take care of my grandparents. I wanted to be like him and support my family.”* (Student 01, Female, Year 1).

This theme suggests that students from close-knit families or disadvantaged backgrounds often see medicine as a way to “give back” to their loved ones and society. Such motivations were deeply personal and often rooted in early life experiences, reinforcing a long-term commitment to patient care.

**Subtheme 1.2: Personal motivation and interest: The intellectual appeal of medicine:** For some students, their interest in medicine was internally driven, stemming from a natural curiosity about science, research, and the human body. These students often cited an early fascination with medical environments, research, and problem-solving as reasons for choosing medicine:

*“I’ve always liked research, discovering new things. Medicine offers endless opportunities to explore.”* (Student 11, Female, Year 2).

*“I like hearing doctors talk about special cases and how they solve them.”* (Student 07, Male, Year 3).

Students who thrived in academic environments and were naturally curious about human biology tended to be more self-motivated in their pursuit of medicine. For them, medicine was not just a means to an end but an intellectually stimulating field that aligned with their desire to engage in lifelong learning. Additionally, previous personal or family illness experiences were a strong motivation for some students:

*“My sister has cerebral palsy, and I felt a responsibility to my family.”* (Student 03, Female, Year 1)

*“I suffered from a dermatological condition that kept recurring. I wanted to understand it better and help myself and others.”* (Student 04, Male, Year 1)

*“As a child, I was stung by a jellyfish and almost didn’t survive. A doctor saved me, and since then, I’ve wanted to be a pediatrician.”* (Student 06, Female, Year 3)

These cases illustrate how personal encounters with illness, either firsthand or through family members, acted as a strong motivator for students, reinforcing their desire to enter the field. This group of students was often more emotionally invested in medicine, viewing it as a personal mission rather than just a career.

**Subtheme 1.3 Family background: Parental expectations and medical exposure:** For some students, the decision to enter medicine was heavily influenced by their family background. This influence could be classified into two subcategories:

**Exposure to the Medical Profession:** Students who had family members in the medical field were more likely to consider medicine a natural career path. Many had parents, grandparents, or relatives working in hospitals, giving them early exposure to the field:

*“My dad is a doctor, and I grew up around medical discussions at home.”* (Student 09, Male, Year 3).

*“My mom runs a small clinic, and I used to interact with patients from a young age. It excited me.”* (Student 08, Female, Year 3).

Such students had an early understanding of what being a doctor entails, making them more confident in choosing medicine. Unlike those driven by social influence or personal illness, these students tended to see medicine as a family tradition rather than an external aspiration.

**Parental Pressure and Expectations:** However, not all students who entered medicine due to family influence did so by choice. Some felt pressured by their parents, who saw medicine as a stable and prestigious profession:

*“My parents forced me to join the medical field because it is stable and respected.”* (Student 10, Male, Year 3).

*“My dad convinced me medicine was the best option for financial security.”* (Student 12, Female, Year 2).

While these students may have entered medical school due to external pressure, their aspirations often evolved over time, either by finding personal fulfillment in medicine or by struggling with a lack of intrinsic motivation.

**Subtheme 1.4. Professional and financial considerations: Prestige and job security:** For some students, medicine was primarily a means to financial stability and societal status. These students were more pragmatic in their decision-making, prioritizing job security over passion:

*“Medicine is highly respected, and it pays well.”* (Student 27, Male, Year 4).

*“Doctors have a good position in society, so it was a logical choice.”* (Student 26, Female, Year 3).

This group often viewed medicine as a stable career path with financial rewards, rather than an emotional calling. Their motivations were primarily extrinsic, and their aspirations were more likely to evolve over time as they encountered the challenges and realities of medical school.

#### Theme 2: Evolution of aspirations throughout medical school.

**Subtheme 2.1. Adjusting to the realities of medical education:** Many students underestimated the workload and difficulty of medical training. The transition from pre-medical idealism to the realities of demanding coursework, hospital rotations, and patient interactions caused some students to reevaluate their initial motivations. For some, the initial passion remained intact but was tested by challenges, while others developed a more practical perspective:

*“Medicine is much harder than I expected. I didn’t think it would be this stressful.”* (Student 25, Male, Year 3).

*“I wanted to do research, but I realized I don’t enjoy it as much as I thought.”* (Student 22, Female, Year 2).

**Subtheme 2.2. Increased focus on work-life balance:** Some students shifted their focus away from intense clinical specializations toward fields with better work-life balance.

*“I used to think I’d do surgery, but I don’t want a career that’s too demanding.”* (Student 23, Male, Year 4).

*“Work-life balance is more important to me now than it was in Year 1.”* (Student 24, Female, Year 4).

This shift was particularly evident among students who initially entered medicine for financial or family reasons, as they became more conscious of the emotional and physical toll of medical practice.

**Subtheme 2.3. Refinement of specialty interests:** Medical school exposed students to a wider range of specialties, leading some to refine or even change their career aspirations. For many, exposure to different medical disciplines helped them identify strengths, weaknesses, and personal preferences, leading to more informed specialty choices:

*“I initially wanted to be a neurologist, but I realized I prefer dermatology.”* (Student 21, Female, Year 3).

*“I don’t enjoy clinical work as much as I thought. Research is more appealing now.”* (Student 20, Male, Year 4).

## Discussion

Medical students’ motivations for pursuing a career in medicine result from a dynamic interplay of intrinsic motivations, extrinsic career considerations, and social/environmental influences. The thematic analysis and quantitative findings indicate that while personal aspirations and altruistic motivations are the primary drivers, students’ career expectations shift as they progress through medical training. These shifts are shaped by clinical exposure, real-world experience, and evolving perceptions of work-life balance, ultimately influencing their specialty preferences.

### Intrinsic motivations: The core of medical aspirations

A strong desire to help people remains the most significant intrinsic motivation among medical students, with newer cohorts increasingly emphasizing patient care (4.08, p = 0.058). This altruistic motivation is often rooted in personal life experiences, such as personal illness or caring for sick family members, which solidify students’ commitment to medicine. The impact of these personal and family health experiences is stable across cohorts (3.42) and is particularly pronounced in those drawn to patient-centered specialties like Pediatrics, OB-GYN, and Psychiatry. In these fields, empathy and compassion are not just beneficial but essential components of medical practice (Supporting information: S1 Table). Supporting this observation, the work of Masmoudi et al. (2024) shows that psychiatry students often report personal psychiatric histories or a family history of mental diseases, indicating a profound personal connection to their specialty [18]. Similarly, a study of genetic counselors revealed that those with personal or family health experiences tend to possess a deeper level of empathy and specialized knowledge, further underscoring the link between personal experiences and professional inclination [19].

As students progress through medical school, their specialty preferences often evolve in response to clinical exposure and self-reflection. While some students remain committed to their initial aspirations, others may shift their focus as they encounter the realities of different medical fields. For example, students who were initially drawn to highly technical or research-intensive fields might later find themselves gravitating towards specialties that involve more direct patient interactions, such as Obstetrics & Gynecology and Pediatrics, or those known for offering a better work-life balance, such as Psychiatry. This evolution in preferences is supported by Hyde’s 2007 study, which found that students preferring direct patient interaction often choose General Practice, OB-GYN, or Pediatrics [20]. Similarly, a 2020 study indicated a generational shift towards prioritizing work-life balance, with over 90% of doctors considering their own capabilities, work-life balance, and personal motivation as critical factors in their career decisions [21]. This trend is corroborated by thematic analysis that highlights how clinical rotations refine students’ interests, aligning their skills with the demands of their chosen specialties.

Additionally, research by Coffeng et al. (2009) emphasizes the importance of the sequence of clerkships in shaping specialty preferences, noting that early clinical experiences, particularly the first clerkship, significantly influence students’ career choices, as these experiences provide a practical context to the theoretical knowledge gained in earlier stages of medical education [22]. These studies collectively demonstrate how both personal inclinations and specialty-based factors dynamically shape medical students’ career paths.

Interest in research and teaching (2.86) shows a modest increase among later cohorts, signaling a growing inclination toward academic medicine, internal medicine, and innovation-driven career paths. However, many students who initially envisioned research-heavy careers later reconsider their choices upon experiencing the rigorous academic demands of medical training, leading some to shift toward clinical specialties such as Internal Medicine or Surgery, which offer intellectual stimulation alongside direct patient care. Studies also reveal various trends. Compared to students in higher class levels, first-year students are reported to be less likely to engage in undergraduate research, while other studies suggest that as students advance in their education, they are less likely to find research useful for their studies compared to first-year students, who were encouraged by professors to explore new interests [23,24]. Commonly mentioned obstacles include a lack of research opportunities and experience, time restrictions, and insufficient opportunities to network with researchers [23,24].

### Extrinsic motivations: Stability and career flexibility

While intrinsic motivations remain dominant, extrinsic factors such as job stability (3.63) and professional opportunities (3.50) moderately influence career choices. The perception of medicine as a secure and adaptable career is reinforced by thematic findings, where students highlight long-term job security and career flexibility as key benefits. However, financial incentives (2.86) and prestige (3.43) do not significantly influence career decisions, suggesting that monetary rewards and societal status alone are not primary motivators. The same pattern was observed in studies among medical students in Mexico and Bahrain, where personal values and specialty content were more determinative than financial reasons and prestige [25,26]. However, opposite trends were found in other studies among medical students in LMICs who prioritize economic and financial concerns when choosing a specialty [27,28].

Geographic background further influences these extrinsic motivations. Students from rural areas place significantly higher importance on job stability (3.72 vs. 3.12, p = 0.022) and financial security (2.98 vs. 2.28, p = 0.016), likely reflecting economic concerns and limited career alternatives outside of medicine (Supporting information: S2 Table). This finding aligns closely with that of Gill et al. (2012), which indicated that prestige and opportunities for research were significantly influential for students with urban backgrounds, while income and positive experiences with clinicians were more influential for students from rural backgrounds [29].

### Social and environmental influences: Limited external pressures

Contrary to common assumptions, external pressures such as family expectations (2.32) and having a doctor in the family (2.25) play a minimal role in shaping career decisions. Thematic analysis confirms that while some students with medical family backgrounds naturally gravitate toward medicine, others experience parental pressure, leading to mixed levels of career satisfaction. Additionally, career guidance (2.66) and social media/movies (2.14) have little influence, reinforcing that students primarily base their career decisions on personal experiences rather than on external portrayals of medicine. Early patient contact and hands-on clinical skills have a greater impact on career choices than media because direct exposure to a specialty provides a more realistic and thorough understanding of the field, helping students make informed decisions [26].

Gender differences also emerge in motivation. Female students place significantly greater emphasis on prestige (3.64 vs. 3.20, p = 0.049), indicating a stronger appreciation for professional recognition (Supporting information: S3 Table). In contrast, male students are slightly more influenced by personal or family illness (3.55 vs. 3.30, p = 0.2), suggesting that direct health-related experiences may be more significant motivators for them. These findings contradict earlier research, which generally suggests that women assign less importance to prestige, exhibit lower career ambitions than their male counterparts, and more commonly prioritize family [5,30]. However, across both genders, financial incentives remain a weak driver, affirming that medical students are more influenced by professional and intrinsic factors rather than economic rewards. As medical students progress through their education, they tend to place greater emphasis on their own well-being and balance their personal and professional lives, rather than focusing solely on financial rewards [31].

### 2. Perceived characteristics of a good doctor and specialty selection

Medical students’ perceptions of what makes a good doctor strongly align with their preferred specialty choices. Attributes such as work ethics, accountability, communication skills, and adaptability play a critical role in shaping specialty preferences.

#### Work ethic and accountability: Suitability for high-intensity specialties.

Students interested in Pediatrics and OB-GYN consider a strong work ethic and accountability crucial due to the demanding nature of these fields (S1 Table). The responsibilities of caring for newborns and mothers, often under urgent conditions, require an exceptional work ethic, with ratings of 5.00 and 4.77 respectively (p = 0.007). Childbirth, intellectual engagement, and the need for continuity of care are significant factors that attract students to these specialties [32]. In contrast, psychiatrists tend to value work-life balance more highly [33]. Nonetheless, challenges such as limited flexibility, psychological stress, and litigation risks in Pediatrics and OB-GYN pose significant recruitment barriers. Enhancing clerkship experiences and promoting better work-life balance in these specialties may help attract more students [32,34].

Similarly, Surgery (4.75, p = 0.021) also demands a stronger work ethic and greater resilience compared to Internal Medicine (S4 Table), as it requires intensive training, long working hours, and complex case management. Unlike patient-centered specialties, surgical careers often attract students who prioritize technical skills, precision, and problem-solving over interpersonal communication. Adaptability also showed a significant difference (p = 0.044), with surgery students rating it higher (4.68) than those in internal medicine (4.22). This reflects the dynamic and often unpredictable nature of surgical procedures, where adaptability is crucial for handling unexpected complications and changes during operations [35].

#### Communication and collaboration: Essential for patient-centered specialties.

Among students opting for surgical specialties (S5 Table), the high score for collaboration (4.63 vs. 4.29, p = 0.006) underscores their appreciation of the multidisciplinary nature of surgical practice. Surgical success depends on effective communication and seamless cooperation among team members, including surgeons, anesthesiologists, nurses, surgical technicians, and other support staff.

Students attracted to these fields recognize the critical role of collaboration skills in managing dynamic and high-pressure surgical situations. Their emphasis on teamwork reflects an understanding that successful surgical outcomes hinge on the team’s ability to work cohesively under demanding conditions. This recognition of the importance of collaborative skills may also guide their career choices, aligning with their competencies and preferences for teamwork in surgical settings [36,37].

#### Problem-solving and adaptability: Managing complex and unpredictable cases.

The assessment of problem-solving and adaptability skills across medical specialties such as Surgery, OB-GYN, and Pediatrics highlights their crucial role in managing complex and unpredictable medical scenarios. These traits are vital for rapid decision-making and adapting to fast-changing conditions, significantly affecting patient care and outcomes.

Students pursuing surgical careers highly value problem-solving skills, scoring an average of 4.70, reflecting the need for sharp decision-making abilities in the high-stakes surgical environment. In contrast, students in Pediatrics and OB-GYN also prioritize problem-solving, though to a lesser extent. This difference likely stems from the unique clinical challenges of these fields, which involve prolonged care and the management of evolving conditions in children and pregnant women.

Adaptability is equally critical, especially in Surgery, where it scores 4.68. Surgical students must adjust their techniques dynamically, indicative of surgery’s unpredictable nature. OB-GYN and Pediatrics also require high adaptability for managing emergencies such as childbirth or acute pediatric conditions, demanding a flexible approach to rapidly changing patient scenarios.

The need for quick problem-solving and adaptability is particularly pronounced in Surgery, OB-GYN, and Pediatrics, where professionals must respond swiftly and effectively to diverse and rapidly evolving medical situations. Building on these findings, studies suggest that medical curricula should include enhanced problem-solving training and simulation-based courses to better prepare students for these demanding specialties [38,39]. Additionally, skill labs and personalized training during clerkships are recommended to improve students’ adaptability and proactive skills [40,41].

#### Implications for policy and practice.

This study provides key insights into medical students’ career aspirations, the traits they value in a good doctor, and how their career goals evolve, offering significant implications for global medical education and workforce planning. However, our recommendations are specific to the context of a new private medical institution in Vietnam and should be interpreted as preliminary:

1. **Career Motivations and Trajectories:** The study underscores the importance of intrinsic motivations, such as altruism, in shaping students’ career choices. Enhancing curricula with elements like patient-centered care and ethics can help sustain interest in crucial but often underserved specialties like family medicine, pediatrics, and psychiatry.
2. **Influence of Doctor Characteristics on Specialty Choice:** Students’ perceptions of essential doctor traits—like empathy, communication skills, and ethical integrity—significantly influence their specialty choices. This suggests a need for educational reforms that tailor teaching methods to develop these competencies, potentially helping balance the distribution of specialists worldwide.
3. **Evolving Career Goals:** The findings reveal that students’ views of the medical profession often start idealized but become more aligned with their personal values and realistic insights through clinical exposure. This evolution highlights the need for dynamic career counseling that adapts to changing student perceptions and provides varied clinical exposure throughout their education.

The study’s findings can inform strategies in medical education systems, especially in regions undergoing transitions. Understanding students’ changing aspirations and specialty preferences allows institutions to tailor curricula that integrate universally valued professional qualities, making medical training more engaging and relevant.

Additionally, insights into how students’ specialty preferences form can help policymakers design targeted incentives to address specialty shortages, particularly in underserved areas like primary care. These strategies should reflect local values and economic conditions to enhance the relevance of medical training and improve retention in local healthcare systems.

Enhanced mentorship programs that provide both professional and personal support can help students navigate their evolving career goals, aligning them with broader healthcare needs. Such adaptive educational strategies ensure that medical students are well-prepared to meet the challenges of modern healthcare environments, both locally and globally.

## Limitations

This study has several limitations to consider when interpreting the findings. First, the sample size, while adequate for a single institution, limits the generalizability to other medical schools, particularly those in different cultural or economic contexts. The reliance on self-reported data may also introduce bias, as students might portray themselves in a more favorable light or in ways they believe align with societal standards. The cross-sectional design restricts the ability to definitively establish causal relationships between students’ motivations and their specialty choices, capturing only a snapshot of attitudes rather than changes throughout their education.

Building upon these initial insights, several avenues for further research are warranted. Longitudinal studies following students throughout their medical education and into their professional careers could offer a deeper understanding of how specialty preferences are shaped over time by clinical exposure, mentorship, and life experiences. Retrospective analyses of practicing physicians could illuminate how initial motivations align, or diverge, from eventual career paths, thereby informing better educational strategies. Moreover, cross-institutional and cross-cultural research involving diverse academic and socioeconomic settings would provide greater generalizability, highlighting context-specific and global trends in medical career decision-making.

## Conclusion

This study elucidates the intricate interplay between intrinsic motivations, such as the desire to help others, and extrinsic factors, such as job stability, in shaping medical students’ specialty choices. It demonstrates that students’ aspirations to study medicine and their views on the qualities of a good doctor significantly influence their specialty decisions. These findings suggest that medical curricula should align more closely with students’ values and the attributes they admire in medical professionals. Such alignment could enhance career satisfaction and better prepare students for the demands of their chosen specialties, thereby addressing global needs in healthcare workforce distribution. Insights from this study are vital for policymakers and educators in optimizing medical training programs to ensure a well-balanced distribution of healthcare professionals across various specialties.

## Supporting information

S1 File Study Questionnaires. (DOCX)

S1 Table Comparative Analysis of Essential Skills and Attributes Across Patient-Centered Specialties. (DOCX)

S2 Table Urban-Rural Differences in Motivations for Pursuing a Medical Career. (DOCX)

S3 Table Gender-Based Differences in Motivations for Pursuing a Medical Career. (DOCX)

S4 Table Perception of Professional and Personal Attributes Across Internal Medicine and Surgical Specialties. (DOCX)

S5 Table Perception of Interpersonal and Communication Skills Across Surgical and Non-Surgical Specialties. (DOCX)

## Abbreviation:

OB-GYN: obstetrics & gynecology
LMICs: low- to middle-income countries
