# Supplementary material for: Aspirations to study medicine, perceptions of a good doctor, and their influence on specialty choice among medical students
Source: PLoS One. 2025 Jun 17;20(6):e0326266. doi: 10.1371/journal.pone.0326266 (PMC12173351; doi:10.1371/journal.pone.0326266)
Supplement: S1 File — (DOCX) [file pone.0326266.s001.docx]

**Medical Students' Career Aspirations, Perceptions of a Good Doctor, and Specialty Choice**

**PART I. QUANTITATIVE STUDY – SURVEY QUESTIONNAIRE**

**Section 1: Demographic Information**

1. **What is your cohort?**
   - Year 1
   - Year 2
   - Year 3
   - Year 4
2. **What is your age?** *(Open-ended response)*
3. **What is your gender?**
   - Male
   - Female
   - Other (please specify)
4. **Where is your permanent residence?**
   - Urban
   - Rural
5. **What is your family's total monthly income?**
   - Less than 10 million VND
   - 10-30 million VND
   - 30-50 million VND
   - More than 50 million VND

**Section 2: Motivations for Choosing Medicine *(Likert Scale: 1-5)***

**To what extent did the following factors influence your decision to pursue medicine?**
*(1 = Not at all, 2 = Slightly, 3 = Moderately, 4 = Very much, 5 = Extremely)*

**Intrinsic Motivations (Personal Aspirations and Interests)**

- Desire to help people
- Interest in research and teaching
- Personal or family illness experience
- High academic achievement in high school

**Extrinsic Motivations (Career Stability and Professional Benefits)**

- Medicine is a prestigious profession
- Stable job security
- High salary potential
- Wide range of career opportunities

**Social and Environmental Influences**

- Having family members who are doctors
- Career guidance from parents or mentors
- Family expectations and pressure
- Influence from social media and movies

**Section 3: Perceptions of a Good Doctor *(Likert Scale: 1-5)***

**To what extent do you think the following characteristics are important for a good doctor?**
*(1 = Not important at all, 2 = Slightly important, 3 = Moderately important, 4 = Very important, 5 = Extremely important)*

**Personal and Ethical Attributes**

- Integrity
- Accountability
- Work ethic
- Humility
- Self-awareness

**Interpersonal and Communication Skills**

- Communication skills
- Empathy
- Compassion
- Collaboration

**Cognitive and Problem-Solving Skills**

- Problem-solving abilities
- Adaptability
- Creativity
- Innovation

**Leadership and Resilience**

- Leadership
- Resilience

**Section 4: Specialty Choice**

**What is your current choice of specialty?** *(Select one)*

- General Medicine
- Surgery
- Pediatrics
- Obstetrics & Gynecology
- Psychiatry
- Dermatology
- Radiology
- Internal Medicine
- Neurology
- Cardiology
- Oncology
- Other (please specify)

**PART II. QUALITATIVE STUDY**

**Interview Guide for Qualitative Study**

This semi-structured interview guide explores how medical students' aspirations evolved and how this influenced their specialty choice.

**Introduction**

- Thank the participants for their time and explain the purpose of the study.
- Ensure confidentiality and obtain consent.

**Section 1: Initial Aspirations and Motivation**

1. Can you share what initially inspired you to pursue medicine?
2. How did your motivations change, if at all, since entering medical school?
3. Were there any personal experiences (e.g., illness, family background) that influenced your decision?

**Section 2: Evolution of Aspirations and Career Decision-Making**

1. Have your career aspirations changed since starting medical school? Why or why not?
2. What factors have influenced these changes (e.g., academic workload, patient interactions, exposure to different specialties)?
3. Have the difficulties of medical school made you reconsider your career path?
4. How have your experiences in medical school influenced your perception of different specialties?
5. Have you developed new interests in medicine that you did not consider before entering medical school?
6. Have you felt any external pressure (family, mentors, societal expectations) in choosing your specialty?
7. How do you think your perception of work-life balance has influenced your specialty preference?
8. Do you feel your initial motivations for studying medicine are still shaping your career choice today?

**Conclusion**

- Is there anything else you would like to add about your journey in medical school?
- Thank the participants and remind them about the confidentiality of their responses.
