# Supplementary material for: Aspirations to study medicine, perceptions of a good doctor, and their influence on specialty choice among medical students
Source: PLoS One. 2025 Jun 17;20(6):e0326266. doi: 10.1371/journal.pone.0326266 (PMC12173351; doi:10.1371/journal.pone.0326266)
Supplement: S1 Table — (DOCX) [file pone.0326266.s002.docx]

**S1 Table: Comparative Analysis of Essential Skills and Attributes Across Patient-Centered Specialties.**

| **Characteristic** | **Obstetrics and Gynecology** | **Pediatrics** | **Psychiatry** | **Overall** | **p-value^1^** |
| --- | --- | --- | --- | --- | --- |
| Work Ethic | 4.77 (± 0.44) | 5.00 (± 0.00) | 3.88 (± 1.13) | 4.61 (± 0.76) | **0.007** |
| Communication Skills | 4.77 (± 0.44) | 4.40 (± 0.84) | 4.50 (± 0.53) | 4.58 (± 0.62) | 0.4 |
| Problem-solving Abilities | 4.54 (± 0.52) | 4.60 (± 0.52) | 4.63 (± 0.52) | 4.58 (± 0.50) | >0.9 |
| Empathy | 4.62 (± 0.65) | 4.40 (± 1.07) | 4.50 (± 0.76) | 4.52 (± 0.81) | >0.9 |
| Resilience | 4.38 (± 0.96) | 4.60 (± 0.70) | 4.38 (± 0.74) | 4.45 (± 0.81) | 0.7 |
| Integrity | 4.54 (± 0.66) | 4.60 (± 0.52) | 4.00 (± 1.07) | 4.42 (± 0.76) | 0.3 |
| Accountability | 4.38 (± 0.77) | 4.50 (± 0.53) | 4.38 (± 0.52) | 4.42 (± 0.62) | 0.9 |
| Collaboration | 4.54 (± 0.52) | 4.30 (± 0.67) | 4.38 (± 0.52) | 4.42 (± 0.56) | 0.6 |
| Compassion | 4.38 (± 0.77) | 4.30 (± 0.67) | 4.13 (± 1.13) | 4.29 (± 0.82) | 0.9 |
| Adaptability | 4.15 (± 0.69) | 4.80 (± 0.42) | 3.88 (± 0.99) | 4.29 (± 0.78) | **0.024** |
| Self-awareness | 4.23 (± 0.73) | 4.50 (± 0.71) | 4.00 (± 1.31) | 4.26 (± 0.89) | 0.6 |
| Humility | 3.85 (± 1.07) | 4.20 (± 0.92) | 3.50 (± 1.20) | 3.87 (± 1.06) | 0.4 |
| Leadership | 3.54 (± 0.78) | 3.60 (± 1.07) | 3.13 (± 1.13) | 3.45 (± 0.96) | 0.6 |
| Creativity | 3.23 (± 0.73) | 3.20 (± 1.23) | 2.75 (± 1.39) | 3.10 (± 1.08) | 0.7 |
| Innovation | 3.15 (± 1.14) | 3.30 (± 1.06) | 2.50 (± 1.20) | 3.03 (± 1.14) | 0.4 |

^1^ Kruskal-Wallis rank sum test
