# Supplementary material for: Aspirations to study medicine, perceptions of a good doctor, and their influence on specialty choice among medical students
Source: PLoS One. 2025 Jun 17;20(6):e0326266. doi: 10.1371/journal.pone.0326266 (PMC12173351; doi:10.1371/journal.pone.0326266)
Supplement: S2 Table — (DOCX) [file pone.0326266.s003.docx]

**S2 Table: Urban-Rural Differences in Motivations for Pursuing a Medical Career.**

| **Characteristic** | **Urban** | **Rural** | **Overall** | **p-value^1^** |
| --- | --- | --- | --- | --- |
| Desire to Help People | 4.04 (± 1.06) | 4.08 (± 1.16) | 4.08 (± 1.14) | 0.6 |
| Stable Job | 3.12 (± 1.33) | 3.72 (± 1.26) | 3.63 (± 1.29) | **0.022** |
| Wide Range of Professional Opportunities | 3.28 (± 1.31) | 3.54 (± 1.19) | 3.50 (± 1.21) | 0.4 |
| Prestigious Profession | 3.36 (± 1.38) | 3.44 (± 1.28) | 3.43 (± 1.29) | 0.9 |
| Illness of Yourself or Close Family/Friend | 3.28 (± 1.06) | 3.45 (± 1.44) | 3.42 (± 1.39) | 0.4 |
| Interest in Research and Teaching | 2.64 (± 1.44) | 2.90 (± 1.32) | 2.86 (± 1.34) | 0.4 |
| Well-paid Job | 2.28 (± 1.34) | 2.98 (± 1.27) | 2.86 (± 1.30) | **0.016** |
| Academic Excellence in High School | 2.56 (± 1.23) | 2.89 (± 1.39) | 2.84 (± 1.37) | 0.3 |
| Career Guidance | 2.52 (± 1.48) | 2.69 (± 1.49) | 2.66 (± 1.49) | 0.6 |
| Family Expectations | 1.88 (± 0.93) | 2.40 (± 1.32) | 2.32 (± 1.27) | 0.093 |
| Doctor Family Background | 2.32 (± 1.57) | 2.24 (± 1.54) | 2.25 (± 1.54) | 0.9 |
| Social media and Movies | 2.04 (± 1.02) | 2.15 (± 1.25) | 2.14 (± 1.21) | >0.9 |

^1^Wilcoxon rank sum test.
