# Supplementary material for: Aspirations to study medicine, perceptions of a good doctor, and their influence on specialty choice among medical students
Source: PLoS One. 2025 Jun 17;20(6):e0326266. doi: 10.1371/journal.pone.0326266 (PMC12173351; doi:10.1371/journal.pone.0326266)
Supplement: S3 Table — (DOCX) [file pone.0326266.s004.docx]

**S3 Table: Gender-Based Differences in Motivations for Pursuing a Medical Career.**

| **Characteristic** | **Male** | **Female** | **Overall** | **p-value^1^** |
| --- | --- | --- | --- | --- |
| Desire to Help People | 4.17 (± 1.07) | 3.99 (± 1.21) | 4.08 (± 1.14) | 0.4 |
| Stable Job | 3.57 (± 1.31) | 3.68 (± 1.28) | 3.63 (± 1.29) | 0.7 |
| Wide Range of Professional Opportunities | 3.55 (± 1.20) | 3.45 (± 1.22) | 3.50 (± 1.21) | 0.6 |
| Prestigious Profession | 3.20 (± 1.38) | 3.64 (± 1.18) | 3.43 (± 1.29) | **0.049** |
| Illness of Yourself or Close Family/Friend | 3.55 (± 1.40) | 3.30 (± 1.37) | 3.42 (± 1.39) | 0.2 |
| Interest in Research and Teaching | 2.95 (± 1.40) | 2.78 (± 1.27) | 2.86 (± 1.34) | 0.4 |
| Well-paid Job | 2.80 (± 1.33) | 2.93 (± 1.28) | 2.86 (± 1.30) | 0.5 |
| Academic Excellence in High School | 2.81 (± 1.39) | 2.86 (± 1.35) | 2.84 (± 1.37) | 0.8 |
| Career Guidance | 2.57 (± 1.52) | 2.75 (± 1.46) | 2.66 (± 1.49) | 0.4 |
| Family Expectations | 2.19 (± 1.29) | 2.44 (± 1.25) | 2.32 (± 1.27) | 0.2 |
| Doctor Family Background | 2.21 (± 1.62) | 2.29 (± 1.48) | 2.25 (± 1.54) | 0.4 |
| Social media and Movies | 2.12 (± 1.21) | 2.15 (± 1.22) | 2.14 (± 1.21) | 0.9 |

^1^Wilcoxon rank sum test.
