# Supplementary material for: Aspirations to study medicine, perceptions of a good doctor, and their influence on specialty choice among medical students
Source: PLoS One. 2025 Jun 17;20(6):e0326266. doi: 10.1371/journal.pone.0326266 (PMC12173351; doi:10.1371/journal.pone.0326266)
Supplement: S4 Table — (DOCX) [file pone.0326266.s005.docx]

**S4 Table: Perception of Professional and Personal Attributes Across Internal Medicine and Surgical Specialties.**

| **Characteristic** | **Internal Medicine** | **Surgery** | **Overall** | **p-value^1^** |
| --- | --- | --- | --- | --- |
| Integrity | 4.22 (± 0.81) | 4.50 (± 0.72) | 4.44 (± 0.75) | 0.13 |
| Empathy | 4.17 (± 1.04) | 4.53 (± 0.83) | 4.45 (± 0.89) | 0.12 |
| Resilience | 4.11 (± 0.96) | 4.63 (± 0.66) | 4.51 (± 0.77) | **0.011** |
| Leadership | 3.50 (± 1.20) | 4.05 (± 0.98) | 3.92 (± 1.05) | 0.074 |
| Compassion | 4.22 (± 1.11) | 4.45 (± 0.79) | 4.40 (± 0.87) | 0.6 |
| Accountability | 4.11 (± 0.90) | 4.48 (± 0.79) | 4.40 (± 0.83) | 0.070 |
| Innovation | 3.61 (± 0.98) | 3.92 (± 1.00) | 3.85 (± 0.99) | 0.2 |
| Adaptability | 4.22 (± 1.00) | 4.68 (± 0.57) | 4.58 (± 0.71) | **0.044** |
| Communication Skills | 4.39 (± 1.14) | 4.63 (± 0.64) | 4.58 (± 0.78) | 0.6 |
| Work Ethic | 4.39 (± 0.92) | 4.75 (± 0.73) | 4.67 (± 0.78) | **0.029** |
| Humility | 3.94 (± 1.21) | 4.17 (± 1.01) | 4.12 (± 1.06) | 0.5 |
| Collaboration | 4.11 (± 0.96) | 4.63 (± 0.61) | 4.51 (± 0.73) | **0.014** |
| Creativity | 3.50 (± 0.99) | 3.92 (± 1.09) | 3.82 (± 1.08) | 0.13 |
| Problem-solving Abilities | 4.39 (± 0.85) | 4.70 (± 0.56) | 4.63 (± 0.65) | 0.11 |
| Self-awareness | 4.28 (± 0.96) | 4.60 (± 0.62) | 4.53 (± 0.72) | 0.2 |

^1^Wilcoxon rank sum test.
