# Supplementary material for: Aspirations to study medicine, perceptions of a good doctor, and their influence on specialty choice among medical students
Source: PLoS One. 2025 Jun 17;20(6):e0326266. doi: 10.1371/journal.pone.0326266 (PMC12173351; doi:10.1371/journal.pone.0326266)
Supplement: S5 Table — (DOCX) [file pone.0326266.s006.docx]

**S5 Table: Perception of Interpersonal and Communication Skills Across Surgical and Non-Surgical Specialties.**

| **Interpersonal and Communication Skills** | **Non-Surgical Specialties** | **Surgical Specialties** | **Overall** | **p-value^1^** |
| --- | --- | --- | --- | --- |
| Communication Skills | 4.43 (± 0.81) | 4.63 (± 0.64) | 4.51 (± 0.75) | 0.10 |
| Collaboration | 4.29 (± 0.82) | 4.63 (± 0.61) | 4.43 (± 0.76) | **0.006** |
| Empathy | 4.36 (± 0.91) | 4.53 (± 0.83) | 4.43 (± 0.88) | 0.2 |
| Compassion | 4.28 (± 0.90) | 4.45 (± 0.79) | 4.35 (± 0.86) | 0.3 |

| ^1^Wilcoxon rank sum test |
| --- |
